# Supplementary figures and images for: Soil Viruses Are Underexplored Players in Ecosystem Carbon Processing
Source: mSystems. 2018 Oct 2;3(5):e00076-18. doi: 10.1128/mSystems.00076-18 (PMC6172770; doi:10.1128/mSystems.00076-18)

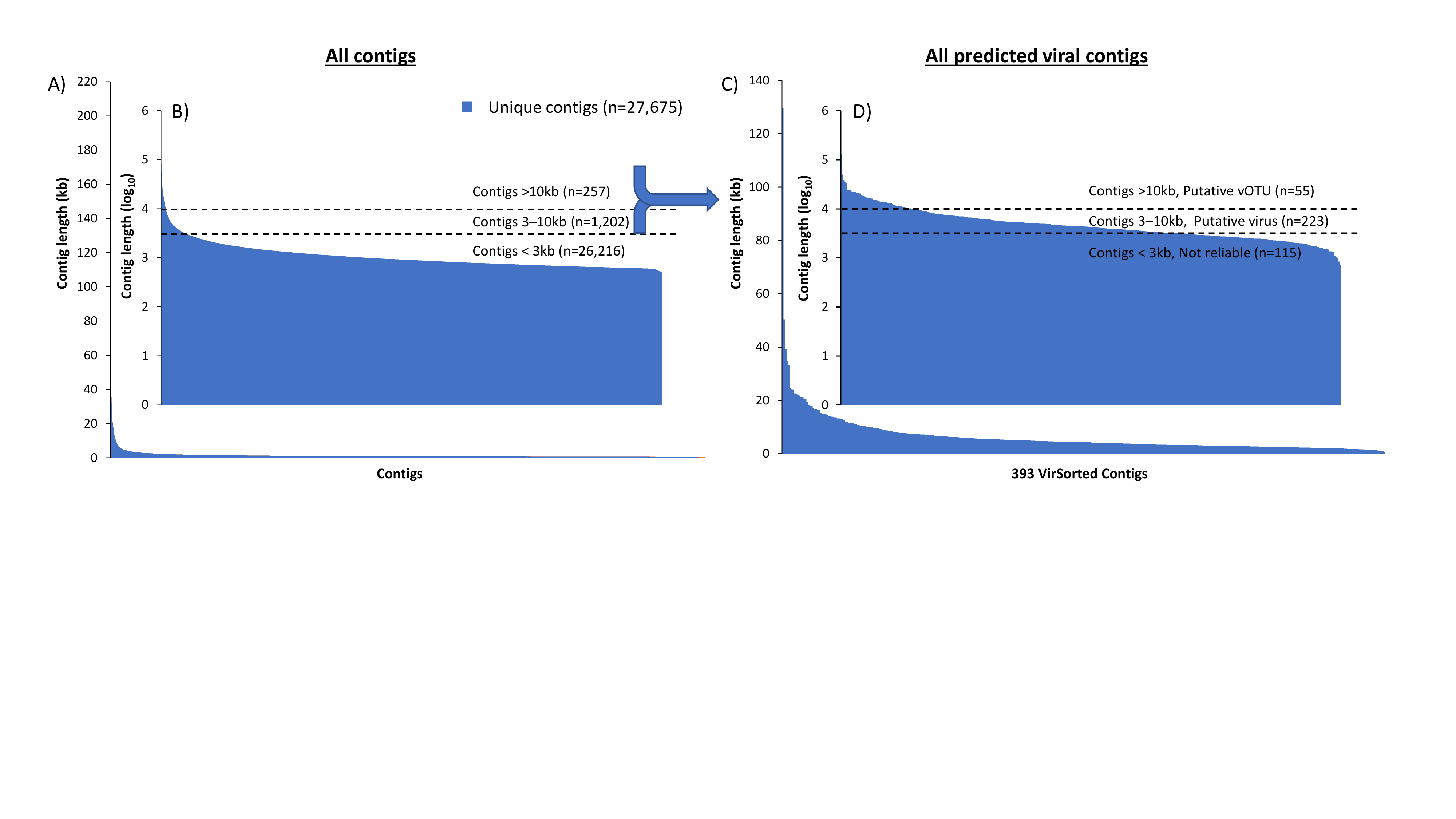

Supplement: FIG S1 [file sys005182265sf1.tif]

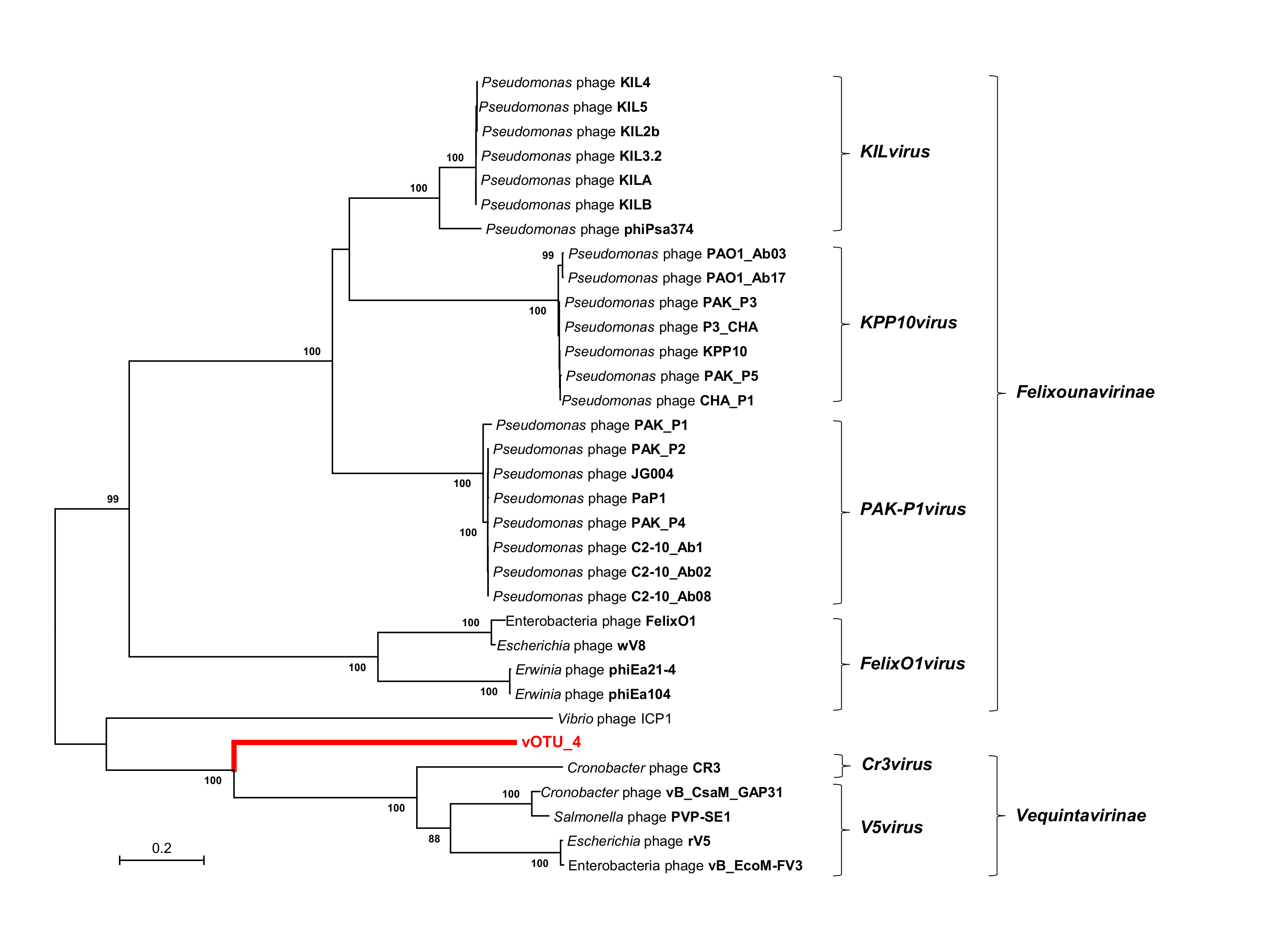

Supplement: FIG S2 [file sys005182265sf2.tif]

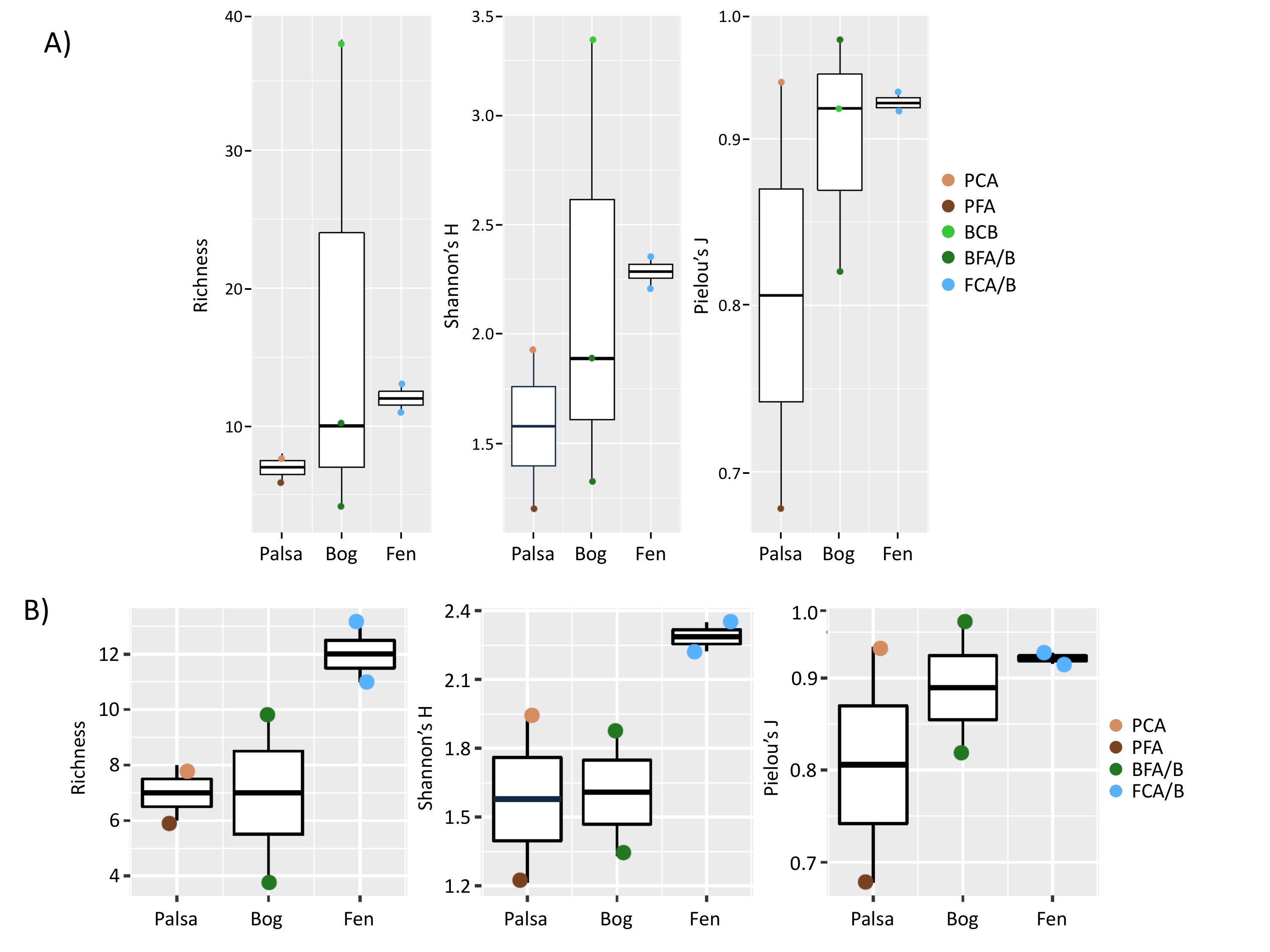

Supplement: FIG S3 [file sys005182265sf3.tif]

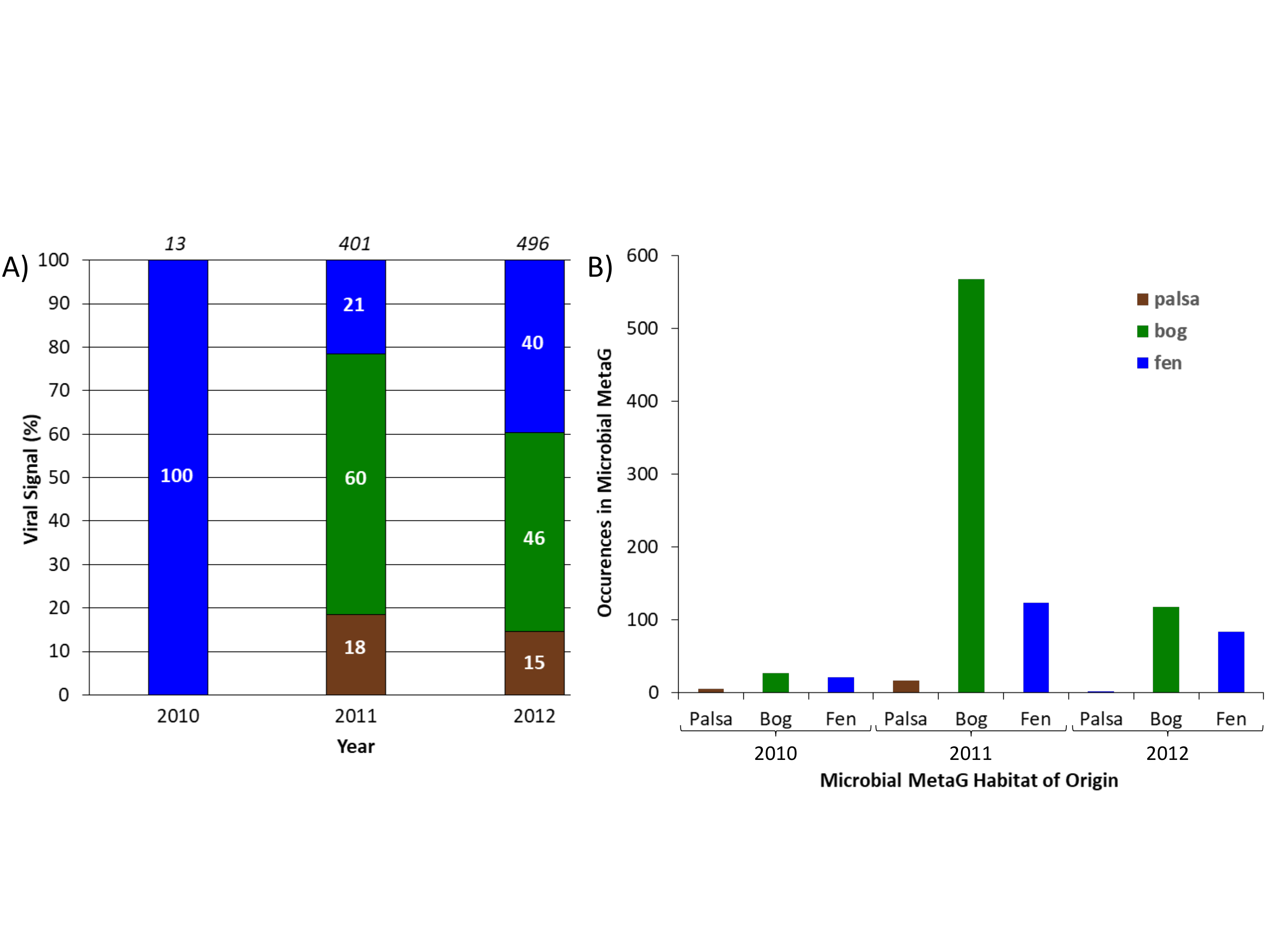

Supplement: FIG S4 [file sys005182265sf4.tif]

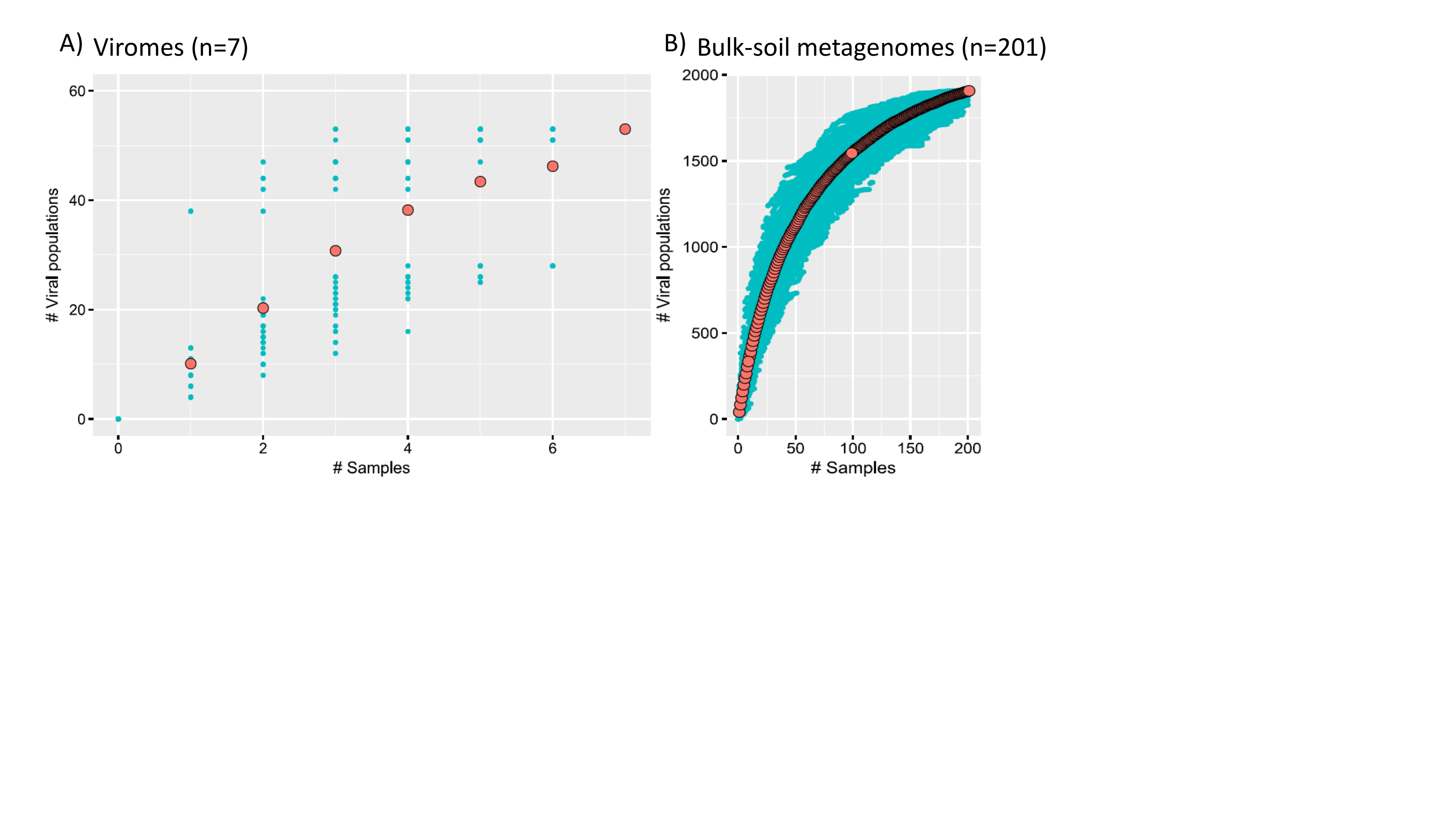

Supplement: FIG S5 [file sys005182265sf5.tif]

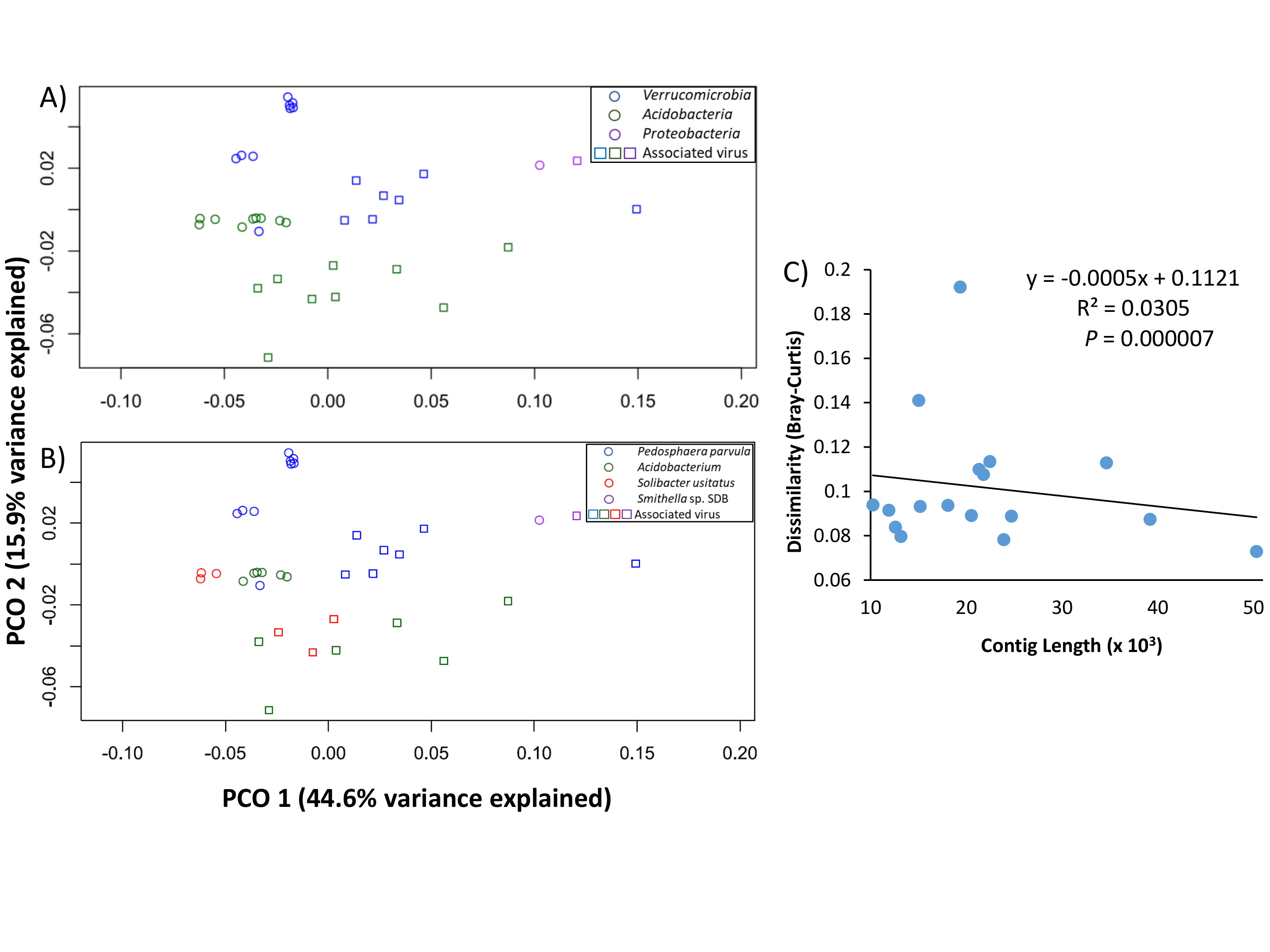

Supplement: FIG S6 [file sys005182265sf6.tif]
