# Supplementary material for: Soil Viruses Are Underexplored Players in Ecosystem Carbon Processing
Source: mSystems. 2018 Oct 2;3(5):e00076-18. doi: 10.1128/mSystems.00076-18 (PMC6172770; doi:10.1128/mSystems.00076-18)
Supplement: TEXT S1 [file sys005182265s1.pdf]

Supplementary text

Addressing overlooked potential contaminants

To aid in identifying possible contamination, we ran a nucleotide Blast of all the assembled contigs against a custom database of the >1500 Stordalen Mire MAGs (for information on the MAGs see 1). No microbial contamination was detected from the microbial lineages represented in the MAGs. Conversely, contamination of GTAs seems likely. The main method used to identify viruses (VirSorter; 2) identifies viruses primarily based on identification of viral genes. GTAs have been confused for viruses or viral contamination because they have several viral-like genes to package nucleic acids (e.g. genes that encode capsids and tails; full list reviewed in 3). We tested for GTAs by assessing the diversity of the contigs identified as microbial (since GTAs capture a subset of lineages) and running a nucleotide Blast of the five types of GTAs previously identified (3–5). In the seven viromes, 25% of assembled reads matched bacteria from the family *Rhodobacteraceae*, and from genera *Bartonella* and *Desulfovibrio*. This analysis likely doesn't capture the total GTA contamination because current GTA families have been identified only from isolates (3). The remaining contigs were divided into those highly likely to belong to soil viruses (as VirSorter categories 1 or 2; 2) and those of indeterminable origin (i.e. VirSorted viral contigs not from categories 1 or 2). Lastly, contamination from mobile genetic elements (MGEs) can result in low viral yield. MGEs are any genetic material that move around on a genome, within an organism, or between organisms (6). Viruses and GTAs are also types of MGEs and others, but not limited to, include transposons, plasmids, and introns. Here, we use MGE to label contamination (i.e. non-viral) and three MGE-looking contigs were identified as viruses by VirSorter (Table S1). It is highly unlikely that these MGEs remained in our samples after CsCl purification and may actually be plasmid prophages (reviewed in 7), but to be as accurate as possible with our findings, we did not include these three contigs. Additionally, we did not include VirSorter category 3 contigs, which are likely to be soil viruses, since the extraction methods were specifically targeted to obtain soil viruses and current reference databases dramatically under-represents soil viruses (causing VirSorter to put them in category 3; more details in 2).

Using viral-host linkages for further comparisons

Since viral replication requires the take-over of host translation machinery during infection, viruses may be genetically tuned to interact with that machinery more efficiently. We searched the 17 host-linked viral genomes for two known mechanisms of such genetic tuning. Viruses can mimic host synonymous codon usage, increasing translation speed and fidelity, level of gene expression, and protein folding (15–17). We assessed the similarity of codon usage frequency for the Mire viruses and their inferred hosts (Fig. S6). The virus-host groups clustered only loosely at the genus/species level (notably better at the phylum level; Fig S6), except for vOTU_28 and its associated host *Smithella* sp. SDB, which were quite similar. This 'codon harmonization' (described in 15) between the two further supports their viral-host CRISPR linkage and inferred co-evolution. Caveats to this approach include the imperfect assessment of viral (and host) codon usage due to incomplete genomes, with the lack of viral single copy genes precluding clarity on how incomplete they are. To assess whether contig length might be

influencing the similarity of virus and host codon usage frequency, we evaluated their correlation (Fig. S6), which was significant but very weak (r^2=0.03, *P* value <0.01), suggesting that longer contigs would have minimal impact on viral-host codon usage similarity.

Putative archaeal viruses

Due to VirSorter's reliance on existing databases, viruses from undersampled environments (e.g. soil) can be missed. Due to the importance of archaea in this system, for example the strong correlation between CH_4 isotopic signatures and a single lineage of methanogens from the Mire (Candidatus *Methanoflorens stordalenmirensis*; 18, 19), we performed an extensive search specifically targeting archaeal viruses using the recently developed Metagenomic Archaeal Virus Detector (MArVD; 20), which retrieves archaeal virus-like contigs from VirSorted-viromes based on a set of genes known to be identified with archaea. Using a stringent threshold (viral contig $\geq$10 kb and belonging to category 1 or 2; 20), MArVD identified 2 putative archaeal vOTUs: 165 and 225, which were ~16 and 12 kb, respectively, but had not been in the 53-vOTU dataset because they did not meet the stringent requirement of being in VirSorter categories 1 or 2 (2). Both vOTUs' genes switch strands often and they were appreciably genetically novel with 83% of genes unknown for vOTU_165 and 75% for vOTU_225, and both had no connectivity to known viruses in the VC network where only three ORFs from each grouped into protein clusters, with the remaining ORFs (15 from vOTU_165 and 19 from vOTU_225) being singletons (proteins having no genetic similarity to any proteins in the RefSeq database). Of the 3 shared PCs, vOTU_165 shared PC_03881 with archaeal virus, Halovirus HCTV-1 (data not shown), which also included archaeal, bacterial, and bacteriophage proteins. Phylogenic reconstruction of PC_03881 revealed monophyly of vOTU_165's gene 16 with genes from *Pseudoalteromonas* (data not shown). Among the other predicted genes from the two putative archaeal viruses, we identified several more for both vOTUs that clustered as bacterial genes, including several ribosomal proteins (data not shown). We therefore propose that both contigs are likely bacterial genome fragments, misidentified by MArVD as it relies on a database with few archaeal representatives and uses keywords to identify archaeal viruses; vOTUs 165 and 225 were identified as potential archaeal viruses based on the identified homologous gene being previously characterized in archaea.

We therefore propose that both contigs are likely bacterial genome fragments, misidentified by MArVD, because the ancestry of PC_03881 (from contig 165) stems from the archaeal domain with a recent transition into bacterial domain (data not shown). This misidentification by MArVD is because it relies on a database with few archaeal representatives and uses keywords to identify archaeal viruses; it identified vOTU_165 and vOTU_225 as potential archaeal viruses based on the identified orthologous gene being previously characterized in archaea.

Methods and Materials

Richness, Shannon's Diversity index and Pielou's evenness index were calculated for each virome in R (CRAN 1.0.8 package vegan).

Multiple analyses were done to evaluate the potential role storage conditions had on the viral communities. Hierarchical clustering of the viromes was done using Bray-Curtis

dissimilarity, with an average dissimilarity used for viromes (i.e. dissimilarities are averaged at each step between viromes for the agglomerative method) in R (CRAN 1.0.8 package pvclust). Assessment of differentiation among reads was performed using Fizkin (a social network analysis; 21) on CyVerse (http://www.cyverse.org) using the default parameters.

More analyses were done to better understand viral-host linkages. For viral-host linkages via CRISPRs, a custom Python script was used to process the Crass data (https://bitbucket.org/MAVERICLab/). Briefly, data Emerson et al. (22) was used to associate viral sequences to microbial bins. First, the assigned taxonomy was parsed and the number of matches to Archaea and Bacteria were summed. If a virus matched a host bin, then this was a "hit" and the number of bins with matches were summed.

Mean synonymous codon usage was calculated by determining the mean of the differences in codon usage frequency for each amino acid for the 55 vOTUs and their linked hosts. Codon usage was determined using CodonW (John Peden, Oxford University, available at http://bioweb.pasteur.fr/seqanal/interfaces/codonw.html) with default parameters.

References

1. Woodcroft, B. J. , Singleton, C. M., Boyd, J. A. , Evans, P. N. , Joanne B. Emerson, Ahmed A. F. Zayed, Hoelzle, R. D., Lamberton, T. O., McCalley, C. K., Hodgkins, S. B. , Wilson, Samuel O. Purvine, Carrie D. Nicora, Changsheng Li, Steve Frolking R. M., Chanton, J. P. , Crill, P. M., Saleska, S. R., Rich, V. I., Tyson, G. W. Genome-centric view of carbon processing in thawing permafrost. Nature, 2018; DOI: 10.1038/s41586-018-0338-1.
2. Roux, S. Enault, F. Hurwitz, B.L. and Sullivan, M.B. 2015. VirSorter: mining viral signal from microbial genomic data. *PeerJ*, *3*, p.e985.
3. Lang, A.S., Westbye, A.B. and Beatty, J.T., 2017. The Distribution, Evolution, and Roles of Gene Transfer Agents (GTAs) in Prokaryotic Genetic Exchange. *Annual review of virology*, *4*(1).
4. Lang, A.S. and Beatty, J.T., 2007. Importance of widespread gene transfer agent genes in α-proteobacteria. Trends in microbiology, 15(2), pp.54-62.
5. Hurwitz, B.L., Hallam, S.J. and Sullivan, M.B., 2013. Metabolic reprogramming by viruses in the sunlit and dark ocean. *Genome biology*, *14*(11), p.R123.
6. Frost, L.S., Leplae, R., Summers, A.O. and Toussaint, A., 2005. Mobile genetic elements: the agents of open source evolution. *Nature Reviews Microbiology*, *3*(9), p.722.
7. Casjens, S., 2003. Prophages and bacterial genomics: what have we learned so far? *Molecular microbiology*, *49*(2), pp.277-300.
8. Kunin, V., Sorek, R. and Hugenholtz, P., 2007. Evolutionary conservation of sequence and secondary structures in CRISPR repeats. *Genome biology*, *8*(4), p.R61.
9. Horvath, P., Romero, D.A., Coûté-Monvoisin, A.C., Richards, M., Deveau, H., Moineau, S., Boyaval, P., Fremaux, C. and Barrangou, R., 2008. Diversity, activity, and evolution of CRISPR loci in Streptococcus thermophilus. *Journal of bacteriology*, *190*(4), pp.1401-1412.
10. Shmakov, S.A., Sitnik, V., Makarova, K.S., Wolf, Y.I., Severinov, K.V. and Koonin, E.V., 2017. The CRISPR Spacer Space Is Dominated by Sequences from Species-Specific Mobilomes. *mBio*, *8*(5), pp.e01397-17.
11. Marraffini, L.A. and Sontheimer, E.J., 2010. Self versus non-self discrimination during CRISPR RNA-directed immunity. *Nature*, *463*(7280), p.568.

12. Kokfelt, U., Rosén, P., Schoning, K., Christensen, T.R., Förster, J., Karlsson, J., Reuss, N., Rundgren, M., Callaghan, T.V., Jonasson, C. and Hammarlund, D., 2009. Ecosystem responses to increased precipitation and permafrost decay in subarctic Sweden inferred from peat and lake sediments. *Global Change Biology*, *15*(7), pp.1652-1663.
13. Olefeldt, D., Roulet, N., Giesler, R. and Persson, A., 2013. Total waterborne carbon export and DOC composition from ten nested subarctic peatland catchments—importance of peatland cover, groundwater influence, and inter-annual variability of precipitation patterns. *Hydrological Processes*, *27*(16), pp.2280-2294.
14. Mondav, R., McCalley, C.K., Hodgkins, S.B., Frolking, S., Saleska, S.R., Rich, V.I., Chanton, J.P. and Crill, P.M., 2017. Microbial network, phylogenetic diversity and community membership in the active layer across a permafrost thaw gradient. *Environmental Microbiology*.
15. Angov, E., Legler, P.M. and Mease, R.M., 2011. Adjustment of codon usage frequencies by codon harmonization improves protein expression and folding. In *Heterologous Gene Expression in E. coli* (pp. 1-13). Humana Press.
16. Butt, A.M., Nasrullah, I., Qamar, R. and Tong, Y., 2016. Evolution of codon usage in Zika virus genomes is host and vector specific. *Emerging Microbes & Infections*, *5*(10), p.e107.
17. Kumar, N., Bera, B.C., Greenbaum, B.D., Bhatia, S., Sood, R., Selvaraj, P., Anand, T., Tripathi, B.N. and Virmani, N., 2016. Revelation of influencing factors in overall codon usage bias of equine influenza viruses. *PloS one*, *11*(4), p.e0154376.
18. Mondav, R., Woodcroft, B.J., Kim, E.H., McCalley, C.K., Hodgkins, S.B., Crill, P.M., Chanton, J., Hurst, G.B., VerBerkmoes, N.C., Saleska, S.R. and Hugenholtz, P., 2014. Discovery of a novel methanogen prevalent in thawing permafrost. *Nature communications*, *5*, p.3212.
19. McCalley, C.K., Woodcroft, B.J., Hodgkins, S.B., Wehr, R.A., Kim, E.H., Mondav, R., Crill, P.M., Chanton, J.P., Rich, V.I., Tyson, G.W. and Saleska, S.R., 2014. Methane dynamics regulated by microbial community response to permafrost thaw. *Nature*, *514*(7523), pp.478-481.
20. Vik, D.R., Roux, S., Brum, J.R., Bolduc, B., Emerson, J.B., Padilla, C.C., Stewart, F.J. and Sullivan, M.B., 2017. Putative archaeal viruses from the mesopelagic ocean. *PeerJ*, *5*, p.e3428.
21. Bolduc, B., Youens-Clark, K., Roux, S., Hurwitz, B.L. and Sullivan, M.B., 2016. iVirus: facilitating new insights in viral ecology with software and community data sets imbedded in a cyberinfrastructure. *The ISME Journal*.
22. Emerson, J.B., Roux, S., Brum, J.R., Bolduc, B., Woodcroft, B.J., Jang, H-B., Singleton, C.M., Solden, L. M., Naas, A. E., Boyd, J. A., Hodgkins, S. B., Wilson, R. M., Trubl, G., Li, C., L., Frolking, S., Pope, P. B., Wrighton, K. C., Crill, P. M., Chanton, J. P., Saleska, S. R., Tyson, G. W., Rich V. I., Sullivan, M. B. Host-linked soil viral ecology along a permafrost thaw gradient. Nature Microbiology, 2018; DOI: 10.1038/s41564-018-0190-y.
23. Daly, R.A., Borton, M.A., Wilkins, M.J., Hoyt, D.W., Kountz, D.J., Wolfe, R.A., Welch, S.A., Marcus, D.N., Trexler, R.V., MacRae, J.D. and Krzycki, J.A. 2016. Microbial metabolisms in a 2.5-km-deep ecosystem created by hydraulic fracturing in shales. *Nature Microbiology*, *1*, p.16146.
24. Pratama, A.A. and van Elsas, J.D., 2018. The 'Neglected'Soil Virome–Potential Role and Impact. *Trends in Microbiology*.
